# Attitudes of undergraduate medical students of Addis Ababa University towards medical practice and migration, Ethiopia

**DOI:** 10.1186/1472-6920-12-68

**Published:** 2012-08-06

**Authors:** Wakgari Deressa, Aklilu Azazh

**Affiliations:** 1 Department of Epidemiology and Biostatistics, School of Public Health, College of Health Sciences, Addis Ababa University, Addis Ababa, Ethiopia; 2 Department of Internal Medicine, School of Medicine, College of Health Sciences Addis, Ababa University, Addis Ababa, Ethiopia

## Abstract

**Background:**

The health care system of Ethiopia is facing a serious shortage of health workforce. While a number of strategies have been developed to improve the training and retention of medical doctors in the country, understanding the perceptions and attitudes of medical students towards their training, future practice and intent to migrate can contribute in addressing the problem. This study was carried out to assess the attitudes of Ethiopian medical students towards their training and future practice of medicine, and to identify factors associated with the intent to practice in rural or urban settings, or to migrate abroad.

**Methods:**

A cross-sectional study was conducted in June 2009 among 600 medical students (Year I to Internship program) of the Faculty of Medicine at Addis Ababa University in Ethiopia. A pre-tested self-administered structured questionnaire was used for data collection. Descriptive statistics were used for data summarization and presentation. Degree of association was measured by Chi Square test, with significance level set at p < 0.05. Bivariate and multivariate logistic regression analyses were used to assess associations.

**Results:**

Only 20% of the students felt ‘excellent’ about studying medicine; followed by ‘very good’ (19%), ‘good’ (30%), ‘fair’ (21%) and ‘bad’ (11%). About 35% of respondents responded they felt the standard of medical education was below their expectation. Only 30% of the students said they would like to initially practice medicine in rural settings in Ethiopia. However, students with rural backgrounds were more likely than those with urban backgrounds to say they intended to practice medicine in rural areas (adjusted OR = 2.50, 95% CI = 1.18-5.26). Similarly, students in clinical training program preferred to practice medicine in rural areas compared to pre-clinical students (adjusted OR = 1.83, 95% CI = 1.12-2.99). About 53% of the students (57% males vs. 46% females, p = 0.017) indicated aspiration to emigrate following graduation, particularly to the United States of America (42%) or European countries (15%). The attitude towards emigration was higher among Year IV (63%) and Internship (71%) students compared to Year I to Year III students (45-54%). Male students were more likely to say they would emigrate than females (adjusted OR = 1.57, 95% CI = 1.10-2.29). Likewise, students with clinical training were more likely to want to emigrate than pre-clinical students, although the difference was marginally significant (adjusted OR = 1.58, 95% CI = 1.00-2.49).

**Conclusions:**

The attitudes of the majority of Ethiopian medical students in the capital city towards practicing medicine in rural areas were found to be poor, and the intent to migrate after completing medical training was found to be very high among the study participants, creating a huge potential for brain drain. This necessitates the importance of improving the quality of education and career choice satisfaction, creating conducive training and working conditions including retention efforts for medical graduates to serve their nation. It follows that recruiting altruistic and rural background students into medical schools is likely to produce graduates who are more likely to practice medicine in rural settings.

## Background

With a population of about 74 million in 2007 [1], Ethiopia is the second most populous sub-Saharan country in Africa next to Nigeria. About 84% of the country’s total populations live in rural areas [1] with the poorest health indicators [2]. The gap between the demand and the provision of basic health services remains substantially high partly due to shortage of trained health workers, particularly physicians [3]. Most of the basic health facilities and health personnel are concentrated in the capital city, Addis Ababa and other regional towns [4].

The Government of Ethiopia and its stakeholders have recently focused on strengthening the health care system through establishing new health facilities and training of physicians and other skilled health workers [5]. The number of medical schools, public hospitals and health centers increased from three, 102 and 583 in 2004/05 [6] to seven, 129 and 2142 in 2009/10, respectively [4]. Similarly, the number of non-governmental organization (NGO) and private hospitals increased from 10 and 19 in 2004/05 [6] to 14 and 51 in 2009/10, respectively [4]. Three private medical schools have been recently established and started training of medical students. Despite the increase in the number of health facilities and medical schools, the health care system suffers from the shortage of and mal-distribution of healthcare workers, particularly physicians.

Human resources for health are one of the most important elements of the healthcare system of any country as the quality of delivering health services depends primarily on the performance of providers. The World Health Organization (WHO) recommends the minimum density of 2.3 doctors, nurses and midwives per 1000 population to achieve the minimum levels of key health interventions [7]. However, the public health sector of many countries in Africa has been facing a serious shortage of physicians, nurses and midwives [8]. In Ethiopia, there were a total of 1,421 physicians (general practitioners and specialists), 3,096 health officers and 26,423 nurses in 2009/10 [4]. The physicians and nurses/midwives per 1000 population were 0.03 and 0.21, respectively [8]. Although the shortages apply to all health personnel, the shortage of physicians remains a significant problem, with one physician serving an average of 56,000 people [3]. As a result, Ethiopia needs additional doctors to meet the health needs of its people, and the ability to train and retain physicians should be the priority of the country to strengthen its health care system.

In Ethiopia, government is the main health care service provider. To address the problem of health care delivery, it is currently striving to improve access to basic preventive and curative health services through expanding physical health infrastructure into rural remote areas and training of various categories of health personnel [5]. In recent years, about 300 medical doctors (general practitioners) graduated from Ethiopian public medical schools each year [4,9]. The strategies mainly focus on meeting the challenges of the health care system for people living in rural and remote areas of the country. However, there has been a continued exodus of physicians from the country [3,8]. From 1987 to 2006, about 73% of medical doctors in Ethiopia left the public sector and migrated to overseas or joined local NGOs/private sector mainly due to attractive payment [3].

Studies indicate that student’s attitudes during their stay in medical school towards training and medical practice can influence their later practice [10-12]. To understand the factors that derive the intentions of practice location and migration among students in health professions, particularly medical students, studies have been conducted in Uganda [12], Nepal [11], West Africa [13], Lebanon [14] and Australia [10]. In addition to the pull and push factors for migration [15-17], researchers have shown a well-developed culture of migration in medical schools [13]. In a study in Nepal, 88% of the medical students thought they would likely to practice in urban areas [11] and most (70%) of the nursing students in Uganda said they would like to work outside the country [12]. Rural background has been shown in many different countries to be a determinant factor associated with medical graduates’ intentions and preferences to practice in rural settings [18-20].

Studies about the intentions and attitudes of medical practice and migration among Ethiopian medical students’ while they are still pursuing their undergraduate training are negligible. Anecdotal evidences show that there are several factors that may influence the attitudes of medical students and their subsequent practice after graduation. Exploring students’ conceptions allows further analysis of the situation to formulate possible interventions that can improve policy at training institutions and at government level. The purpose of this study was to assess the attitudes of Ethiopian medical students training in the capital city towards their training and future practice of medicine, and to identify factors associated with the intent to practice in rural or urban settings, or to migrate abroad.

## Methods

Undergraduate medical students at the Faculty of Medicine (FoM) at Addis Ababa University (AAU) participated in the study conducted in June 2009. Formal training of medical professionals in Ethiopia began in 1964 with the opening of the FoM at AAU with its tertiary teaching hospital (Tikur Anbessa) in the capital city, Addis Ababa. The Faculty has been considered as the pioneer medical school in Ethiopia, followed by Gondar and Jimma medical schools. The FoM at AAU had 19 departments at the time of the study and admits students as per the national criteria set by the Federal Ministry of Education based on the performance of secondary school leaving examinations. Although students either from free government secondary schools or private fee-based schools from all parts of the country join the FoM, the majority come from private or NGO high schools, which predominate in Addis Ababa or other larger cities in the country.

New students joining the FoM at AAU initially attend general courses for six months in the pre-medical training program before joining pre-clinical education during the following Year I and Year II. All medical students completing pre-clinical and clinical (Year III and Year IV) trainings are required to do a one year internship program. In general, the duration for undergraduate medical training has a curriculum lasting for 5½ years, and 2–4 years postgraduate training. Until the end of 2006, the FoM has graduated 1890 general medical practitioners (MD) and 862 specialists in various fields [3].

This cross-sectional study based on quantitative data collection method involved all medical students (Year I to internship program) at the FoM. In June 2009, the total number of full-time undergraduate medical students at AAU from Year I to internship program was 800 and about 36% of them were females. A self-administered pre-tested structured English questionnaire was used for data collection. English is the medium of instruction in all higher teaching institutions in Ethiopia. Most of the questions were closed-ended with pre-coded responses, mainly grouped into socio-demographic characteristics and attitudes towards medical practice and migration. Some questions used a Likert scale indicating the extent to which students agreed or disagreed to the statements. Based on their training background, students were divided into two main categories: (i) pre-clinical (Year I and II) and (ii) clinical (Year III, Year IV and internship). The questionnaires were administered after gathering the students in the lecture halls. Instructions on how to properly fill the questionnaire, particularly on how to follow the skip patterns, were given to the students.

The data were entered into Epi Info version 6.04d and analyzed using SPSS version 16 software (SPSS Inc, Chicago, IL, USA). Descriptive statistics were used for data summarization and presentation. Degree of bivariate associations was measured using the Pearson Chi Square test to assess the significance between different variables, with significance level set at p < 0.05. Finally, a multivariate analysis was done by fitting the logistic regression model to identify factors associated with intention to initially practice medical profession in rural areas and perceptions of emigration by controlling for the effect of potential confounding variables such as age, sex, religion, rural–urban background, parental education and sources of family’s income. Adjusted odds ratios (ORs), along with 95% confidence intervals (CI), were reported.

Ethical approval was received from the Institutional Review Board of the FoM at AAU. Responsible people in the Faculty were informed about this study. Participation of the students was voluntary and the purpose of the study was explained to them prior to the distribution of the questionnaires. Any identification of the students was not recorded anywhere on the questionnaire and appropriate measures were also taken to ensure confidentiality of information.

## Results

Of 800 registered undergraduate medical students at the FoM, 632 participated in filling the self-administered questionnaires, yielding a response rate of 78%. The main reason for non-participation was the unavailability of students at the time of the survey due to clinical practices in hospitals in Addis Ababa. Of 632 filled questionnaires, 600 were used for final data analysis; the remaining (5%) was rejected due to incompleteness. Of the total participants, 72% were pre-clinical and 28% were clinical with regard to their medical education status.

The detail of the demographic characteristics of the study participants was published elsewhere [21]. Briefly, the mean age of respondents was 20 years with standard deviation of 2, and 69% were males. The age distribution and the mean age were similar for both male and female students. About 61% of the study participants attended high school in Addis Ababa. With regard to religion, the overwhelming majority (63%) belonged to Orthodox Christianity, followed by Protestant Christian (20%) and Muslim (14%). The majority (80%) of the students were from urban backgrounds compared to 20% of the students from rural backgrounds. About 32% of the respondents reported that their fathers attained first degree or above and 15% of them reported that their mothers attained similar level of education. Similarly, 31% of the students reported that their parents were government employees, 26% reported that the main source of family’s income was business, 22% of the family’s income was based on agriculture and 12% of them reported that their parents were NGO/private firm employees.

Table 1 shows the motivations for choosing medicine as a field of study, and fewer than half chose medicine because they were interested in the field or in life saving activities (40% males vs. 60% females, p < 0.001). About one in five of the overall students reported they chose medicine for better income (25% males vs. 11% females, p < 0.001). More males (16%) than females (8%) chose medicine for social prestige. About 11% of the students reported they chose medicine due to family or peer influence, while less than 2% felt the choice was not theirs. With regard to their current feelings towards studying medicine, about 20% of the respondents said ‘excellent’ (20% males vs. 19% females), 19% rated it as ‘very good’, and 30% rated it as ‘good’. However, 32% of them (33% males and 31% females) felt either ‘fair’ or ‘bad’ towards studying medicine. About 20% of the students (22% males vs. 15% females, p = 0.024) stated that medicine was not the right field of study for them and 34% (41% males vs. 20% females, p < 0.001) reported they suffered from financial problem while in the medical school.

**Table 1:** AAU medical students’ reported reasons for choosing medicine as a field of study and current feelings toward the discipline by gender

**Characteristics**	**Gender**		**Total, n (%)**	**p-value**
	**Male, n (%)**	**Female, n (%)**		
**Main reason for choosing medicine**				
Interested in the field or in life saving	160 (39.5)	116 (59.5)	276 (46.0)	**<0.001**
For better income	102 (25.2)	22 (11.3)	124 (20.7)	**<0.001**
Social prestige	65 (16.0)	15 (7.7)	80 (13.3)	**0.005**
Family or peer influence	43 (10.6)	20 (10.3)	63 (10.5)	0.893
Had no better choice	5 (1.2)	5 (2.6)	10 (1.7)	0.395
Assigned or imposed	7 (1.7)	2 (1.0)	9 (1.5)	0.761
God’s will or childhood dream	4 (1.0)	3 (1.5)	7 (1.2)	0.855
Other	19 (4.7)	12 (6.2)	31 (5.2)	0.448
**Current feelings towards studying medicine**				
Excellent	81 (20.0)	36 (18.5)	117 (19.5)	0.656
Very good	72 (17.8)	40 (20.5)	112 (18.7)	
Good	119 (29.4)	58 (29.7)	177 (29.5)	
Fair	80 (19.8)	47 (24.1)	127 (21.2)	0.928
Bad	53 (13.1)	14 (7.2)	67 (11.2)	**0.031**
**Total, N (%)**	**405 (67.5)**	**195 (32.5)**	**600 (100)**	

Note: P-values in bold are statistically significant (p < 0.05).

Source: A survey conducted in June 2009 among 600 Addis Ababa University medical students in Ethiopia.

Table 2 presents respondent’s ratings of their expectation of medical education and the role of instructors. About 35% of respondents (35% males vs. 37% females) responded they felt the standard of medical education was below their expectation. The majority of the respondents (62%) agreed their instructors were role models for them, although 37% were equivocal about it. More than 13% of respondents reported their instructors exhibited poor attendance at lectures and practical sessions.

**Table 2:** AAU medical students’ reported opinion about their expectation of medical education, instructors’ roles model and rate of attendance of instructors during lectures and practical sessions by gender

	**Gender**		Total, n (%)*	**p-value**
	**Male, n (%)**	**Female, n (%)**		
**Agreement on medical education to the standard of the respondent’s expectation**				
Completely agree	30 (7.5)	9 (4.6)	39 (6.6)	0.504
Agree	87 (21.8)	34 (17.5)	121 (20.4)	0.225
Somewhat agree	144 (36.1)	79 (40.7)	223 (37.6)	0.275
Disagree or completely disagree	138 (34.6)	72 (37.1)	210 (35.4)	0.546
**Agreement on instructors as a role model for medical students**				
Completely agree	79 (19.8)	41 (21.1)	120 (20.2)	0.704
Agree	172 (43.1)	79 (40.7)	251 (42.3)	0.581
Somewhat agree	111 (27.8)	64 (33.0)	175 (29.5)	0.195
Disagree or completely disagree	37 (9.3)	10 (5.2)	47 (7.9)	0.082
**Rate about the attendance of instructors during lectures and practical sessions**				
Excellent	103 (25.8)	59 (30.4)	162 (27.3)	0.238
Very good	127 (31.8)	74 (38.1)	201 (33.9)	0.127
Good	109 (27.3)	43 (22.2)	152 (25.6)	0.177
Fair	46 (11.5)	16 (8.2)	62 (10.5)	0.221
Bad	14 (3.5)	2 (1.0)	16 (2.7)	0.081
**Total, N (%)**	**399 (67.3)**	**194 (32.5)**	**593 (100)**	

*Data missing for 7 participants.

Source: A survey conducted in June 2009 among 600 Addis Ababa University medical students in Ethiopia.

We asked participants about where they would like to initially practice after graduation (Table 3). For place of initial employment, about 30% (95% CI = 25.9-33.3) said they would like to practice in rural areas of the country after completing their training (30% males vs. 28% females, p = 0.501), while 28% preferred to work in urban areas (26% males vs. 32% females, p = 0.151). However, 21% of the participants (23% males vs. 17% females, p = 0.089) said they would prefer to work abroad following graduation, without serving in the country. A great number of participants (44%) reported their preference was to initially practice medicine in public sector compared with NGOs (17%) or private sector (6%). For postgraduate training, the overwhelming majority (79%) of respondents (78% males and 80% females) said they would prefer clinical medicine to biomedical sciences research (7%) or public health (6%).

**Table 3:** AAU medical students’ reported place of initial practice of medicine and organization as well as area of specialization upon graduation by gender

**Characteristics**	**Gender**		**Total, n (%)**	**p-value**
	**Male, n (%)**	**Female, n (%)**		
**Place to initially practice medical profession**				
Rural areas	123 (30.4)	54 (27.7)	177 (29.5)	0.501
Urban/city	106 (26.2)	62 (31.8)	168 (28.0)	0.151
Abroad	93 (23.0)	33 (16.9)	126 (21.0)	0.089
Any where	78 (19.3)	40 (20.5)	118 (19.7)	0.717
Other	5 (1.2)	6 (3.1)	11 (1.8)	0.211
**Organization in which to initially practice medicine**				
Public sector	177 (43.7)	85 (43.6)	262 (43.7)	0.971
Any organization/institution	62 (15.2)	43 (22.1)	105 (17.2)	0.042
NGOs	66 (16.3)	35 (17.9)	101 (16.8)	0.612
Organization in abroad	63 (15.6)	18 (9.2)	81 (13.5)	**0.034**
Private sector	27 (6.7)	9 (4.6)	36 (6.0)	0.322
Other	10 (2.5)	5 (2.6)	15 (2.5)	0.834
**Area of specialization to pursue**				
Clinical medicine	316 (78.0)	156 (80.0)	472 (78.7)	0.580
Biomedical sciences	34 (8.4)	9 (4.6)	43 (7.2)	0.093
Public health	28 (6.9)	10 (5.1)	38 (6.3)	0.400
Other	27 (6.7)	20 (10.3)	47 (7.8)	0.125
**Total, N (%)**	**405 (67.5)**	**195 (32.5)**	**600 (100)**	

Note: P-values in bold are statistically significant (p < 0.005).

Source: A survey conducted in June 2009 among 600 Addis Ababa University medical students in Ethiopia.

Socio-demographic and behavioral factors associated with intention to initially practice medicine in rural areas are presented in Table 4. The variables were initially assessed by bivariate analysis and retained in the multivariate models whether or not they were statistically significant. Among nine factors included in the multiple logistic regression models, variables such as older age, rural background and clinical medical training were significant predictors of intention to initially practice medical profession in rural areas. The odds to initially practice medicine in rural areas of the country were 1.8 times higher among older students (aged 20 or more years) compared to those 19 or younger students (adjusted OR = 1.83, 95% CI = 1.12-3.01). Only 18% of students aged less than 20 compared to 36% of those aged 20 or more would like to initially practice medicine in rural areas.

**Table 4:** Socio-demographic and other correlates of intention to practice medicine in rural areas in the country among AAU medical students

**Factors**	**Intended to initially practice medicine in rural areas**		Adjusted OR* (95% CI)	**p-value**
	**No, n (%)**	**Yes, n (%)**		
**Gender**				
Female	141 (72.3)	54 (27.7)	1.00 (Reference)	
Male	282 (69.6)	123 (30.4)	0.95 (0.62, 1.47)	0.823
**Age in years**				
15–19	177 (82.3)	38 (17.7)	1.00 (Reference)	
20 or more	246 (63.9)	139 (36.1)	1.83 (1.12, 3.01)**	**0.017**
**Religion**				
Other Christian	82(63.1)	48 (36.9)	1.00 (Reference)	
Orthodox Christian	273 (73.2)	100 (26.8)	**0.60 (0.38, 0.95)**	**0.030**
Muslim	60 (72.3)	23 (27.7)	0.59 (0.31, 1.16)	0.130
Other	8 (57.1)	6 (42.9)	0.83 (0.25, 2.72)	0.754
**Completed high school in Addis Ababa**				
No	160 (69.3)	71 (30.7)	1.00 (Reference)	
Yes	263 (71.3)	106 (28.7)	1.38 (0.80, 2.37)	0.247
**Original background**				
Urban	358 (74.3)	124 (25.7)	1.00 (Reference)	
Rural	65 (55.1)	53 (44.9)	2.50 (1.18, 5.26)**	**0.016**
**Medical education status**				
Pre-clinical	319 (74.5)	109 (25.5)	1.00 (Reference)	
Clinical	104 (60.5)	68 (39.5)	1.83 (1.12, 2.99)**	**0.017**
**Mother’s educational level**				
Never attended school	118 (62.1)	72 (37.9)	1.00 (Reference)	
Elementary or high school	96 (75.6)	31 (24.4)	0.77 (0.41, 1.47)	0.432
Above 12^th^ grade	209 (73.9)	74 (26.1)	0.82 (0.40, 1.66)	0.576
**Father’s educational level**				
Never attended school	85 (59.9)	57 (40.1)	1.00 (Reference)	
Elementary or high school	93 (74.4)	32 (25.6)	0.60 (0.32, 1.12)	0.107
Above 12^th^ grade	245 (73.6)	88 (26.4)	0.82 (0.40, 1.68)	0.585
**Main source of family’s income**				
Government employee	138 (74.6)	47 (25.4)	1.00 (Reference)	
Business	120 (75.5)	39 (24.5)	0.84 (0.47, 1.48)	0.539
Agriculture-based	73 (58.4)	52 (41.6)	0.95 (0.40, 2.26)	0.913
Other	92 (70.2)	39 (29.8)	0.99 (0.58, 1.71)	0.978
**Total, N (%)**	**423 (70.5)**	**177 (29.5)**		

*OR = Odds Ratio, **An OR >1 indicates greater likelihood of practicing in a rural area compared with practicing in urban.

Note: P-values in bold are statistically significant (p < 0.05).

Source: A survey conducted in June 2009 among 600 Addis Ababa University medical students in Ethiopia

Among several variables, original background (rural vs. urban) appeared to play a major role in location decisions after graduation; there were greater odds of students with rural background to initially practice medicine in rural areas compared to those with urban background (adjusted OR = 2.50, 95% CI = 1.18-5.26) (Table 4). About 45% of the students from rural background compared to 26% of those from urban background intended to initially practice medicine in rural areas. Students in clinical training preferred to practice medicine in rural areas compared to pre-clinical students (adjusted OR = 1.83, 95% CI = 1.12-2.99). About 40% of clinical students compared to 26% of pre-clinical students reported to work in a rural area.

**Figure 1:**
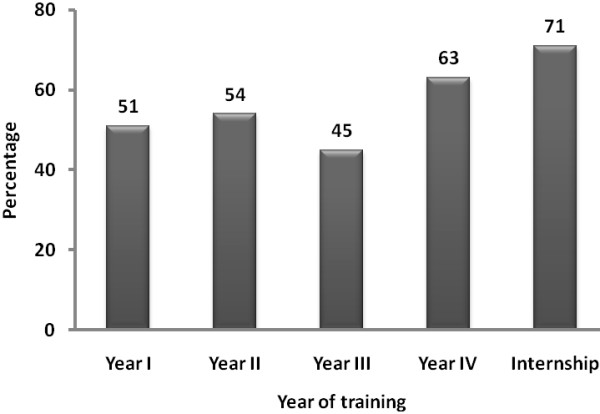
Pattern of intention to emigrate outside Ethiopia upon graduation.

Overall, 53% (95% CI = 49-57) of the students participated in the study expressed an intent to emigrate out of Ethiopia following graduation (57% males vs. 46% females, p = 0.017) (Table 5). Of students who expressed intention to emigrate, 42% were attracted towards the United States (US) of America; followed by emigration to European countries (15%) or Canada (5%). About 2% of the students also preferred to migrate to Botswana, the only country cited in Africa for emigration and 6% preferred to migrate to any country that offers better payment (4% males vs. 10% females, p = 0.047). About 3% of the students intended to emigrate to any country other than Ethiopia and about 18% have not yet decided where to emigrate (16% males vs. 24% females, p = 0.084). The intent of the students to migrate out of Ethiopia after graduation was substantially higher among Year IV (63%) and Internship (71%) students compared to Year I to Year III (45-54%) students (Figure 1).

**Table 5:** Intention to emigrate after graduation and commonly cited destination countries among AAU medical students by gender

	**Gender**		**Total, n (%)**	**p-value**
	**Male, n (%)**	**Female, n (%)**		
**Preferred to emigrate and work abroad after graduation (n = 600)**				
Yes	229 (56.5)	90 (46.2)	319 (53.2)	**0.017**
No	176 (43.5)	105 (53.8)	281 (46.8)	
**Total, N (%)**	**405 (67.5)**	**195 (32.5)**	**600 (100)**	
**Commonly cited countries where to emigrate after graduation (n = 319)**				
United States of America	102 (44.2)	33 (37.5)	135 (42.3)	0.282
European countries	38 (16.5)	9 (10.2)	47 (14.7)	0.161
Canada	12 (5.2)	4 (4.5)	16 (5.0)	0.961
Botswana	5 (2.2)	1 (1.1)	6 (1.9)	0.886
Any country that pays better	10 (4.3)	9 (10.2)	19 (6.0)	**0.047**
Any country other than Ethiopia	6 (2.6)	3 (3.4)	9 (2.8)	0.989
Other countries	22 (9.5)	8 (9.1)	30 (9.4)	0.906
Not yet decided	36 (15.6)	21 (23.9)	57 (17.9)	0.084
**Total, N (%)**	**231 (72.4)**	**88 (27.6)**	**319 (100)**	

Note: P-values in bold are statistically significant (p < 0.05).

Source: A survey conducted in June 2009 among 600 Addis Ababa University medical students in Ethiopia.

To explore factors associated with intent to migrate upon graduation, multiple logistic regression analysis was performed to estimate odds ratios using nine independent variables (Table 6). Gender is the only notable variable positively associated with intention to emigrate; males were more likely to emigrate than females (adjusted OR = 1.57, 95% CI = 1.10-2.29). Students with clinical training were marginally more likely to emigrate following graduation than pre-clinical students although the difference was not statistically significant (adjusted OR = 1.58, 95% CI = 1.00-2.49). Age of respondents, region, original background, parent’s educational level and family’s source of income did not appear to predict intention to emigrate following graduation.

**Table 6:** Socio-demographic and other correlates of intention to emigrate after graduation among AAU medical students

**Factors**	**Intended to emigrate**		Adjusted OR* (95% CI)	**P-value**
	**No, n (%)**	**Yes, n (%)**		
**Gender**				
Female	105 (53.9)	90 (46.2)	1.00 (Reference)	
Male	176 (43.5)	229 (56.5)	**1.57 (1.10, 2.29)**	**0.020**
**Age in years**				
15–19	95 (44.0)	121 (56.0)	1.00 (Reference)	
20 or more	186 (48.4)	198 (51.6)	0.69 (0.46, 1.06)	0.088
**Religion**				
Other Christian	67 (51.5)	63 (48.5)	1.00 (Reference)	
Orthodox Christian	173 (46.3)	201 (53.7)	1.27 (0.83, 1.92)	0.268
Muslim	35 (42.2)	48 (57.8)	1.54 (0.84, 2.81)	0.161
Other	6 (46.2)	7 (53.8)	1.68 (0.52, 5.50)	0.388
**Completed high school in Addis** **Ababa**				
No	102 (44.5)	127 (55.5)	1.00 (Reference)	
Yes	179 (48.3)	192 (51.7)	0.70 (0.44, 1.11)	0.129
**Original background**				
Rural	60 (51.3)	57 (48.7)	1.00 (Reference)	
Urban	221 (45.8)	262 (54.2)	1.25 (0.63, 2.46)	0.527
**Medical education status**				
Pre-clinical	206 (48.2)	221 (51.8)	1.00 (Reference)	
Clinical	75 (43.4)	98 (56.6)	1.58 (1.0, 2.49)	0.051
**Mother’s educational level**				
Never attended school	96 (50.8)	93 (49.2)	1.00 (Reference)	
Elementary or high school	48 (37.8)	79 (62.2)	1.57 (0.88, 2.79)	0.127
**Father’s educational level**				
Never attended school	73 (51.4)	69 (48.6)	1.00 (Reference)	
Elementary or high school	50 (40.3)	74 (59.7)	1.33 (0.75, 2.37)	0.331
Above 12^th^ grade	158 (47.3)	176 (52.7)	0.97 (0.51, 1.84)	0.921
**Main source of family’s income**				
Government employee	81 (44.3)	102 (55.7)	1.00 (Reference)	
Business	75 (47.2)	84 (52.8)	0.83 (0.51, 1.35)	0.445
Agriculture-based	65 (52.0)	60 (48.0)	0.79 (0.36, 1.73)	0.560
Other	60 (45.1)	73 (54.9)	1.04 (0.65, 1.68)	0.868
**Total, N (%)**	**281 (46.8)**	**319 (53.2)**		

*OR = Odds Ratio, **An OR >1 indicates greater likelihood of practicing in a rural area compared with practicing in urban.

Note: P-values in bold are statistically significant (p < 0.05).

Source: A survey conducted in June 2009 among 600 Addis Ababa University medical students in Ethiopia.

## Discussion

The main aim of this study was to identify factors associated with medical students’ perception of their career choice, satisfaction with their education, and intentions to practice medicine. The study revealed mixed feelings of the students towards low levels of satisfaction related to their career choice and quality of medical education. In this study we found that about one-third of the students were dissatisfied with studying medicine and felt the standard of medical education was below their expectation. Furthermore, about one-third of them were equivocal about the role model and class attendance of their instructors during lectures and practical sessions.

Quality of education and career choice satisfaction are the main factors associated with the success of medical training. Poor learning and working conditions for the students and faculty may be one of the factors that discourage them with their learning-teaching processes. The fact that medical profession is a prestigious choice, studies indicate that medical students have reservations about medicine as a career [22]. Students’ attitudes toward medical profession and practice can be influenced during training, and these attitudes can affect their intentions of practice after graduation. In Uganda, poor working conditions and inadequate compensations were the major factors affecting job satisfaction and morale of health workers [23]. Similar factors are also affecting job satisfaction and morale of faculty at medical schools, particularly in low-income countries like Ethiopia [3]. Faculty may be discouraged with inadequate payment at public medical schools and engaged in private works to get better income, which can result in poor preparedness and dissatisfaction towards teaching. Improving students’ expectations through better learning-teaching processes, improving working conditions, updating of curricula and staff development are needed to counteract the decline in faculty attitudes and students’ expectations toward medical career and practice.

In our study, about 44% of the medical students expressed a preference to practice in the public sector than the private sector (6%) upon graduation. The public sector is the main employer of health workers in Ethiopia, and most of the new graduates of medicine face an obligation to serve the public to compensate for their training expenses. It has become usual that most of the experienced medical specialists and even general practitioners move to the private sector and NGOs following the completion of the obligation mainly due to low payment levels in the public sector. For example, 66% of clinical specialists and 85% of general practitioners were lost from public health institutions in Ethiopia between 1987 and 2006 [3]. The migration of skilled health professionals from public to private or NGO sector in the country is similar with that of other countries in sub-Saharan Africa [24]. Consequently, the public sector suffers from a shortage of experienced and skilled health professionals.

Similar to our findings, the overwhelming majority of students in health profession in Uganda preferred to initially practice in the public sector over the private sector [12]. The main reason for health professionals to stay in the public sector may be for future training, academic aspect and gaining experience before joining the private sector [25]. A study in South Africa indicated that private sector nurses were more satisfied than those in the public sector due to their low payment, workload and shortage of resources at the later [26]. Other studies also indicate that achievements, remuneration and job attributes are the main motivators of health care professionals [27-29].

We found 30% of medical students in our study would like to initially practice medicine in rural areas after graduation, while 20% were willing to serve anywhere. Most junior medical practitioners in the country start their career in rural areas and then gradually move to the most urbanized areas, leading to a high staff turnover and under staffing in rural and remote areas. The Ministry of Health reported that 43% of the physicians (general practitioners and clinical specialists) in the country worked in Addis Ababa in 2009/10 [4], where only 3.7% of the population resides. The medical doctors profile study in the country also revealed similar pattern of accumulation of medical doctors in Addis Ababa [3].

In Hungary, the majority of the young doctors preferred to work in large cities or major teaching/central hospitals due to high salary, availability of professional standards, satisfactory working environment, access to skilled colleagues and modern equipment [30]. A study conducted in Uganda indicated that 80% of the nursing students preferred to work in urban than rural areas [12], which was higher than our findings. Likewise, 40% of the medical and nursing students in Australia showed a preference for working in large urban centers within one year, but would consider moving to a more rural location later in life [10].

We found 40% of clinical students compared to 26% of pre-clinical students preferred to initially practice medicine in rural areas of the country. Exposure to rural areas through rural attachment may be one reason why students’ intentions to work in rural areas was higher for clinical students over pre-clinical students. In the FoM at AAU, medical students undertake a rural community based training for six weeks at the 4^th^ year during clinical training. A study from Australia also indicated that rural placements in the undergraduate health training programs have a predominantly positive influence on students’ intention to work in rural areas after graduation [31,32]. A similar study also revealed that exposure of urban background students to rural practice generates an interest among the students to serve in rural areas [33]. Studies affirm the importance of rural training as part of a strategy to maintain and build a rural health professional workforce [32-34]. A training program consisting of clinical skills, community health, practice management and communication skills for newly placed rural doctors in Mali significantly contributed to their retention in rural areas [34].

We found that students with rural backgrounds are more likely to practice medicine in rural areas over students with urban backgrounds. Various literatures identified rural background as the strongest variable associated with the retention of health professionals in rural communities [18-20,34]. In part, this association can be explained by the familiarity that rural background students have with rural setting and cultural norms. We also identified students aged 20 or more preferred to initially practice medicine in rural settings. Studies confirm the substantial changes in attitude and maturity of medical students towards clinical and ethical practice during the course of medical education [35].

The findings from this study indicate that 53% of the medical students intended to migrate out of Ethiopia after completing their medical school training, and the intent increased from pre-clinical to internship. In Uganda, most (70%) of the nursing students would like to work outside the country [12]. Similarly, 52% of Nepali medical students said they would like to practice medicine abroad [11]. Emigration of health care professionals from low- and middle-income countries to high-income ones is becoming a serious global agenda, negatively affecting the health systems of source countries [15,36-38].

Large numbers of African–trained physicians migrate to high-income countries upon completion of their medical school training [16,39]. Our findings indicate that 42% of those who intended to migrate cited US as a final destination, followed by European countries (15%). More than 265 physicians in the US and Canada in the year 2002 were originally trained in Ethiopia and the number of physicians remaining at home for the same year was 1564 [40], representing 15% of Ethiopia’s potential medical workforce without even considering those who migrated to other countries. Of Ethiopian physicians who lived in the US or Canada in 2002, 75% of them were graduates of AAU medical school. About 23-28% of physicians in the US, UK, Canada and Australia are international medical graduates mostly coming from low-income countries, and Ghana, South Africa, Ethiopia, Uganda and Nigeria are the countries in sub-Saharan Africa with the highest physician emigration factors in the world [41].

One limitation of this study is that the sample consisted of more pre-clinical students than clinical ones and, therefore, may have had a less proportion of students who were exposed to a rural training program or to senior colleagues, resulting in underestimation of the intention to practice medicine in rural areas or to migrate outside Ethiopia. This is because of the recent government initiatives to increase the intake of students into medical schools in the country. The other limitation of the study was the low response rate of clinical students compared to pre-clinical ones due to the engagement of the former in practicum trainings at different hospitals, underestimating the responses than the typical. Finally, this study was based on self-reported data and possibly affected by a recall bias. However, all the above limitations may not have a detrimental effect on the validity of the findings, and data can be used for planning cost-effective and sustainable interventions to bring about positive changes in the attitudes of medical students towards medical practice and migration.

## Conclusions

This paper is among the first to study medical students perception of medical practice and emigration in Ethiopia. We conclude that a number of factors are associated with the attitudes of students towards medical practice and migration. Only one-third of the students said they preferred to practice medicine in rural areas of the country upon graduation and more than half of them intended to emigrate after completing medical school training. The study suggested that students with rural backgrounds were more likely to want to practice in rural areas than students with urban backgrounds. Being male was highly associated with intention to emigrate than females.

### Recommendations

A range of activities should be targeted to improve the quality of education and career choice satisfaction, to shape the attitudes of medical students to practice in rural settings and discourage the culture of medical migration. Furthermore, improving the quality of faculty and their attendance should be a priority, so that students will think of them as appropriate role models. Exposure to rural training program can foster positive attitudes to rural practice and encourage medical students to practice in rural areas after graduation [32-34,42,43]. Our findings have also important policy implications that the number of students with a rural background enrolled into medical schools should be increased to produce graduates who are more likely to practice in rural settings and attention should be given at recruiting more altruistic students into medical schools. In addition to competitive salaries, retention efforts after graduation must also focus on the provision of economic incentives, better professional opportunities and satisfactory working conditions in order to decrease dissatisfaction and minimize brain drain.

## Competing interests

The authors declare that they have no competing interests.

## Authors’ contributions

WD involved in proposal writing, designing the study and in all implementation stages of the project. He did most of the analysis and write up of the paper. AA conceived the original idea, involved in proposal writing and in all stages of the project implementation and write up. All authors read and approved the final manuscript.

## Pre-publication history

The pre-publication history for this paper can be accessed here:

http://www.biomedcentral.com/1472-6920/12/68/prepub
